# Hemosiderosis is associated with increased susceptibility to *Yersinia pseudotuberculosis* infection in Seba’s short-tailed bats (*Carollia perspicillata*)

**DOI:** 10.1177/03009858251343017

**Published:** 2025-06-18

**Authors:** Simon Spiro, Alexander Griffiths, Ahmad Arnaout, Ethan Wrigglesworth, Shaheed Karl Macgregor, Shinto Kunjamma John, Stamatios Alan Tahas, Emma Nye, Alexander P. Morrell

**Affiliations:** Zoological Society of London, London; Department of Earth Science and Engineering, Imperial College London, London, UK; London Metallomics Facility, Research Management & Innovation Directorate, King’s College London, London; Zoological Society of London, London; Zoological Society of London, London; Zoological Society of London, London; Copenhagen Zoo, Roskildevej 32, Frederiksberg 2000, Denmark; Experimental Histopathology, The Crick Institute, London, UK; London Metallomics Facility, Research Management & Innovation Directorate, King’s College London, London

**Keywords:** Yersinia pseudotuberculosis, hemosiderosis, iron, bats, inductively coupled plasma mass spectrometry, laser ablation

## Abstract

*Yersinia pseudotuberculosis* (Yptb) is a gram-negative bacterium that can cause sporadic, fatal infections in humans, domestic animals and wildlife. We describe an outbreak of Yptb in a captive collection of 222 Seba’s short-tailed bats (*Carollia perspicillata*), 50 of which died of confirmed (39/222, 17.6%) or suspected (11/222 5.0%) Yptb infection. Females were more likely to be infected than males (odds ratio 3.4) and non-pregnant females were much more likely to be infected than pregnant females (odds ratio 13.6). The most common gross lesions were multifocal cream/white discolorations and/or nodules (30/39, 77%) in the liver, followed by splenomegaly (23/39, 59%) and mesenteric lymphadenomegaly (9/39, 23%); 5/39 (13%) animals had no gross lesions. Histopathology was performed on the livers of 33 confirmed Yptb^+^ animals, with the most common findings being extramedullary hematopoiesis (27/33, 82%) and pyogranulomatous or suppurative hepatitis (20/33, 61%). Hemosiderosis was observed in 32/33 (97%) of cases and in 27/27 (100%) of control animals that were not infected with Yptb. Solution inductively coupled mass spectrometry showed that infected bats had an average of 1.7x more hepatic iron than uninfected bats (p=0.0067); this was corroborated by image analysis of Perl’s stained sections (p<0.0001) but laser ablation on a subset of cases was not significant (p=0.1051). We hypothesize that hemosiderosis favors the systemic spread of Yptb by limiting the efficacy of hepcidin-mediated iron depletion and that limiting dietary iron may protect captive wildlife from bacterial infections.

*Yersinia pseudotuberculosis* (Yptb) is a motile, gram-negative rod within the order *Enterobacteriales*.^2^ Although closely related to *Yersinia pestis*, the causative agent of plague,^1^ Yptb only causes rare, food-borne disease (yersiniosis) in humans, usually presenting as gastroenteritis and mesenteric lymphadenitis,^37^ though progression to fatal septicemia is reported.^10^ Fatal infection is more common in animals, with the disease reported in domestic animals such as cats, sheep, goats and pigs ^67^ and in a wide variety of zoo animals including primates, artiodactyls, rodents, shrews, parrots and toucans.^15,28,33,38,47^ Fatal epizootics of Yptb have also been reported in captive colonies of Egyptian fruit bats (*Rousettus aegypticus*) and Seba’s short-tailed bats (*Carollia perspicillata*).^17,32,46^

Iron is the most ubiquitous element on Earth and is an essential nutrient for all animal life, playing roles in electron transport, oxygen transport, cell proliferation and the regulation of gene expression.^8^ Iron is similarly vital for microorganisms, so infectious agents such as bacteria must have ready access to iron to proliferate inside a host.^4,75,78^ This results in competition between pathogen and host for available iron; hosts continuously work to reduce free iron by sequestering it in carrier molecules such as transferrin and lactoferrin^77^ while pathogens acquire iron from these molecules either by expressing host-specific receptors for them or by competitively acquiring iron using siderophores.^31,56^ During infection, hosts further deplete serum iron to inhibit the growth of pathogens, such as by IL-6 induced hepatic synthesis of hepcidin, the major iron regulatory hormone, which favors the uptake of iron into cells.^48^ Intracellular iron is further sequestered by increased synthesis of ferritin, each molecule of which can sequester up to 4500 iron atoms.^58^ Chronic inflammation results in extended hypoferremia and limits the availability of iron for erythropoiesis, recognized clinically as anemia of chronic disease.

The importance of iron scavenging to *Yersinia* species has been long recognized. Non-pigmented colonies of *Yersinia pestis* are less virulent in mice than the parent, pigmented strain, and virulence can be restored by injecting mice with non-toxic doses of hemin.^12^ The non-pigmented strain arises via spontaneous deletion of a 102 kb high pathogenicity island termed *pgm*; this encodes genes for a siderophore, yersiniabactin, and genes for the storage of hemin, which is the source of the pigmentation.^5,6,25,26,29^ Yersiniabactin is well conserved across *Yersinia* species, including Yptb.^14^ EV76 is an effective plague vaccine derived from non-pigmented strains,^62^ though it has not been approved by regulators due to safety concerns.^43,69^

If iron restriction can inhibit the establishment of infection by *Yersinia* species, the corollary arises of whether iron overload can enhance it. In 2011, a microbiology researcher died of septicemic plague caused by an attenuated, non-pigmented strain of *Yersinia pestis*. Post mortem examination (PME) and genetic testing demonstrated that the researcher had undiagnosed hereditary hemochromatosis (HH).^27^ In mouse models, HH restores the virulence of non-pigmented *Y. pestis* ^54^ and enhances the colonization of deep tissues by Yptb.^44^ HH is caused by the C282Y mutation in the *HFE* gene, resulting in excess dietary absorption of iron; between 5-10% of North Europeans carry the allele with up to 1/100 being homozygous.^42^ Numerous case reports exist of humans with HH suffering from hepatic or septic disease from Yptb or *Y. entercolitica* infections, even though these pathogens usually only cause gastroenteritis, suggesting HH facilitates bacterial colonization beyond the gut.^39,63,70,72^

The terminology around iron storage diseases is inconsistently used across the literature; this report will use the most common veterinary definitions wherein “hemosiderosis” is used to refer to any amount of excessive iron storage and “hemochromatosis” is reserved for heavy iron deposition causing liver damage.^68^ Early onset hemochromatosis has been reported in certain animal species, including Salers cattle and red deer, and appears to be genetic, though neither has the mutation seen in human HH.^50,52^ Hemosiderosis is commonly reported in a wide variety of captive wildlife, including species of cetaceans, chiroptera, passeriformes, perissodactyls, piciformes, pinnipeds, primates and xenarthans. ^3,9,24,30,40,53,66,74^ While there is likely some degree of genetic susceptibility, diet is one of the most important factors in the development of hemosiderosis.^18^ Browser rhinoceros species do not show hemosiderosis in the wild, but regularly develop it in captivity, presumably due to the evolution of highly-efficient iron absorption in the face of a naturally iron depleted diet; in the black rhinoceros (*Diceros bicornis*) an S88T polymorphism in the HFE gene has been implicated.^7,49^ Hemosiderosis of captive wildlife is largely an incidental finding at PME, but it can occasionally progress to hemochromatosis, especially in older animals.^18,21,30,49^

As both hemosiderosis and yersiniosis are common in captive wildlife, it is possible that the former increases susceptibility to the latter, as it does in humans and experimental mouse models.^12,44,76^ Previous attempts to examine this hypothesis have been retrospective examinations of multiple disease outbreaks, involving multiple species of host and bacterium, and/or have not had an objective way of quantifying the level of hemosiderosis.^15,19,36^ In this study, we report a large outbreak of Yptb in a colony of Seba’s short-tailed bats (*Carollia perspicillata*) that also had a high incidence of hemosiderosis. The extensive and prolonged nature of the epizootic, the risk of spread to adjacent enclosures containing endangered species and the risk to the welfare of the bats necessitated management euthanasia (ME) of the whole enclosure, providing a natural experiment wherein any association between hemosiderosis and susceptibility to Yptb infection could be investigated. In addition to traditional histochemical techniques, spatial quantification of iron in histological sections was also performed at 5 µm resolution using laser ablation inductively couple plasma mass spectrometry (LA-ICP-MS), and the techniques compared. We hypothesize that bats with natural Yptb infection (Yptb^+^) will have higher hepatic iron concentrations than ME bats without Yptb (Yptb^-^).

## Materials and Methods

### Animals

All animals were part of the live collection of the Zoological Society of London (ZSL) London Zoo. All bats either died naturally or were humanely euthanized with isoflurane due to disease or for population management. No animals were euthanized solely for the purposes of this, or any other, scientific research.

### Pathological investigations

A full gross post-mortem examination (PME) was performed on each bat that died or was euthanized with clinical signs of disease. A full set of tissues was preserved in 10% neutral buffered formalin, and samples of liver were frozen at -20°C.

ME animals received an abbreviated post-mortem examination in which they were weighed, the elbow-carpus length was measured and the external surfaces of all coelomic organs were examined *in situ*; the brain was not examined. If this process detected suspicion of disease, a full PME was performed. If the uterus was enlarged, it was incised, and the fetus was externally examined. Separate samples of liver were preserved in 10% neutral buffered formalin and frozen at -20°C, and the liver was swabbed for Yptb culture as below.

### Microbiology

Sterile swabs were taken from the cut surface of the liver of every animal, with the exception of some cases early in the outbreak and cases where the carcasses were substantially autolyzed or mummified. Swabs were inoculated onto 5% horse blood agar (Oxoid Ltd, Basingstoke, UK) and incubated aerobically, CO_2_ anaerobically and in microaerophilic conditions, at 37°C, for up to 5 days or until colonies consistent with Yptb were seen, whichever was sooner. Colonies were speciated as *Enterobacteriaceae* based on Gram stain & colony morphology, and a negative oxidase reaction. Yptb was then confirmed with a 99.4 to 99.9% probability based on analytical profile index 20E (BioMerieux, Basingstoke, UK). If no colonies were isolated after 5 days, samples were considered negative; if *Proteus mirabilis* overgrew the plate then samples were considered inconclusive.

### Image analysis

The 32 best-preserved livers from infected (Yptb^+^) bats, and 27 livers from randomly selected, sex matched, uninfected (Yptb^-^) bats were processed into formalin-fixed paraffin embedded sections, using standard protocols. Consecutive 4 µm sections were stained with hematoxylin and eosin (H&E) and with Perl’s Prussian blue. All Perl’s staining was performed simultaneously using the same batches of chemicals and incubation times. Stained sections were scanned using an Axio Scan Z1 (ZEISS, Oberkochen, Germany) at 20x magnification and were read digitally using NDP View 2 (Hamamatsu Photonics, Hamamatsu City, Japan). All Perl’s-stained slides were scanned in the same run using identical exposure settings.

The livers of five Yptb^+^ bats were autolyzed, with dissociation of hepatocyte cords and excessive background; histological description of these livers was performed but they were excluded from all subsequent iron quantification investigations. Three 1 mm^2^ regions of interest (ROI) were selected from each liver, avoiding areas of necrosis, autolysis, tissue edges and large vessels and bile ducts. These regions were selected on H&E-stained sections, by an author with no histopathology training (AA), to avoid bias in selecting areas low or high in hemosiderin. These ROI were converted to the JPEG file format. ImageJ^64^ was used to divide the image into 8-bit red, green and blue channels. Tissue (i.e. non-background) was selected by adjusting the threshold to 178, and Perl’s-positive pixels were selected by subsequently adjusting the threshold to 87. ImageJ’s “analyze particles” function was then used to quantify the percentages of the total ROI area that was composed of tissue and hemosiderin; data were then expressed as the percentage of hemosiderin area by whole tissue area.

### Inductively coupled plasma mass spectrometry

Frozen sections of liver were placed in pre-weighed, acid-cleaned, trace-metal grade HDPP 15 ml centrifuge tubes (Elkay Laboratory Products, Basingstoke, UK) and dehydrated by heating to 70°C overnight. The tubes were then re-weighed and the dry mass calculated. The samples were incubated at room temperature in 0.15 ml Optima grade concentrated H_2_O_2_ (30-32% w/w, Sigma Aldrich, St Louis, USA). The samples were further incubated in an additional 0.45 ml Optima grade HNO_3_ (67-69% w/w, Sigma Aldrich, St Louis, USA) overnight until completely digested. The weight of the digested material was calculated and 0.1 µg of gallium standard was added to each tube. Iron concentration by dry weight was then determined by inductively coupled plasma mass spectrometry (ICP-MS).

### Laser ablation ICP-MS

Unstained FFPE sections were dewaxed in three consecutive, five-minute baths of 100% ultrapure xylene (Sigma Aldrich, St Louis, USA) and three consecutive, one minute baths in 100% ethanol (Sigma Aldrich, St Louis, USA), all with manual agitation, and air dried. Brightfield microscopy was used to identify the ROI previously selected for IA, using section edges and large vessels as landmarks.

An Iridia 193 nm ArF*excimer-based laser ablation (LA) system (Teledyne Photon Machines) was used, equipped with the cobalt long-pulse ablation cell. The LA system was coupled to a ThermoFisher Scientific iCAPTQ ICP-MS (ThermoFisher Scientific) by means of the Aerosol Rapid Introduction System. Full operational parameters for both the Iridia and iCAPTQ ICP-MS are provided in Supplementary Table 1. Tuning of the instrument settings was performed using a NIST SRM 612 glass certified reference material (National Institute for Standards and Technology), optimizing for low laser-induced elemental fractionation by monitoring 238U^+^/232Th^+^, oxide formation rates (<1%) through the 232Th16O^+^/232Th^+^ ratio and the sensitivity of 59Co^+^, 115In^+^ and 238U^+^. LA-ICP-MS image was acquired in a fixed dosage mode, with a vertical and horizontal spatial resolution of 5 μm. Samples were mounted on a four-slide sample holder of the cobalt cell (Teledyne Photon Machines). The selected isotope of interest, ^56^Fe, was chosen to maximize sensitivity whilst minimizing isobaric/polyatomic interferences and increasing signal-to-noise ratio. Dynamic Reaction Collision (DRC) mode with oxygen as the reaction gas was employed in the collision reaction cell (CRC) to reduce the contribution of polyatomic interferences on imaging. To correct for instrumental drift, a series of NIST 612 standard ablation scans were performed before and after experimental samples. Quantitative imaging was achieved using gelatine micro droplet standards sourced from the Institute of Analytical Chemistry, University of Vienna.^65^

ICP-MS and positional data were reconstructed to generate elemental images using the HDF-based Image Processing software (HDIP, Teledyne Photon Machines Inc., Bozeman, MT, USA). A bespoke in-house pipeline, written in Python (version 3.9), was used to analyze the reconstructed data and produce comparable elemental images. The pipeline consisted of removing negative values, attributed to instrumental noise, and replacing with zeros. All data is presented on the same intensity scale for comparability (95^th^ percentile).

### Statistics

Data were collected and stored using Excel 365 (Microsoft Corporation, Redmond, USA) and all statistical analyses were performed using GraphPad Prism version 9.5.0 (Dotmatics, Boston, USA). Column analyses were performed using a two-tailed Mann-Whitney U test. Trendlines were calculated using least-squares regression and compared using the extra sum-of-squares F test. To produce histograms of the LA-ICP-MS results, the base-10 logarithm of each iron concentration reading (in µg/g) was calculated in Excel 365 and a frequency distribution was produced with bin-widths of 0.001 in GraphPad Prism. Odds ratios were calculated using Fisher’s exact test. Quoted error is always standard deviation unless otherwise stated.

## Results

### Outbreak profile

ZSL London Zoo maintained a colony of approximately 200 Seba’s short-tailed bats from January 2010 until June 2020. Over the 10-year period from January 2010 to December 2019, 106 adult bats died or were euthanized due to clinical disease, an average of less than 1 per month. In that time, mortality only exceeded 3/month on three occasions; there were four deaths in January 2010 due to transport stress, 7 and 17 in December 2011 and January 2012 respectively due to an outbreak of Yptb and 22 in February 2014 following a stressful catch-up event. Thus, there was concern when five adult bats were found dead over a 4-day period in March 2020.

Bacterial cultures from the livers of affected animals quickly identified Yptb as the etiological agent. Over the subsequent 12 weeks, a further 40 adults died and three were euthanized after being found collapsed and dyspneic; four infections were detected in animals euthanized during depopulation, giving a total of 52 suspect cases out of a total adult population of 222 (23%). Of these 52 cases, hepatic microbiology was performed on 44, of which 34 were confirmed positive for Yptb. Of the ten cases that tested negative or inconclusive, five had histological and/or gross evidence of yersiniosis and so were categorized as confirmed case. Two animals had no lesions consistent with Yptb infection on gross or histological examinations: one had cardiomegaly and hepatic congestion, and so was considered more likely to have died incidentally of heart failure, while the other remained undiagnosed. Both were excluded from the study. Of the remaining 11 cases, nine were too autolyzed for examination and two had no gross or histological lesions but had not been swabbed for microbiology; these were all considered possible/undiagnosed. Thus, the study population comprised 39 confirmed cases (Yptb^+^),11 possible/undiagnosed cases and 170 control animals (Yptb^-^), all of which were management euthanasias with negative liver cultures. Details of all bats are provided in Supplemental Table 2.

**Figure 1:**
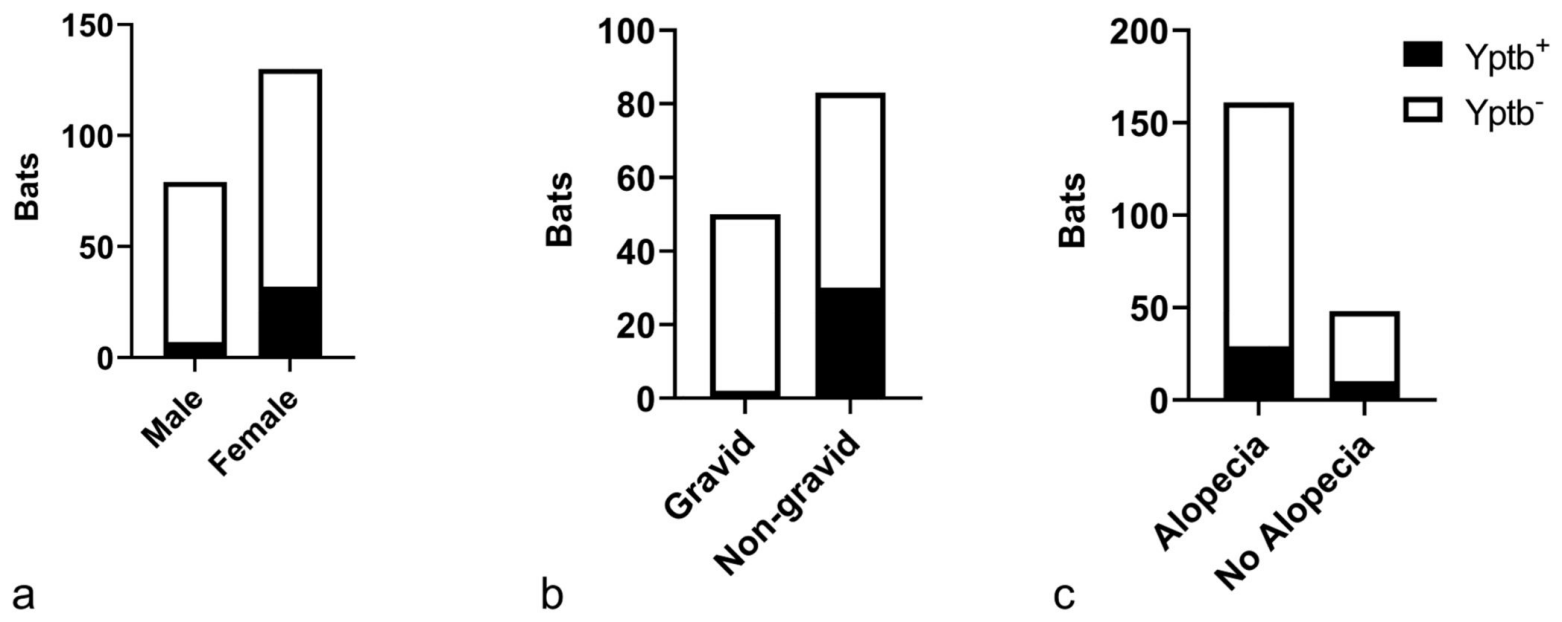
Post mortem findings in Seba’s short-tailed bats (*Carollia perspicillata*) infected (Yptb+) and uninfected (Yptb-) with *Yersinia pseudotuberculosis*. (**a**) Female bats were 3.4x more likely to be Yptb+ than male bats. (**b**) Yptb+ bats were 13.4x less likely to be pregnant than Yptb- bats. (**c**) There was no significant association between alopecia and Yptb infection status.

There was a considerable sex-bias associated with Yptb infection. In the Yptb^+^ population, 32/39 were female (82%); in the Yptb^-^ population 98/170 (58%) bats were female (Fig. 1a). This gives an odds ratio of 3.4 for female bats to be Yptb^+^ compared to male bats (p=0.0055, Fisher’s exact test). Furthermore, pregnancy was much more common in Yptb^-^ (48/101, 48%) than Yptb^+^ females (2/32, 6%), an odds ratio of 13.6 (p<0.0001, Fisher’s exact test, Fig. 1b). The bats commonly had some degree of alopecia of the ventral thorax: 29/39 (74%) in Yptb^+^ and 132/170 (78%) in Yptb^-^ bats, indicating no statistically significant association between the two conditions (p=0.6753, Fisher’s exact test, Fig. 1c).

### Gross and microscopic lesions of yersiniosis and haemosiderosis

**Figure 2:**
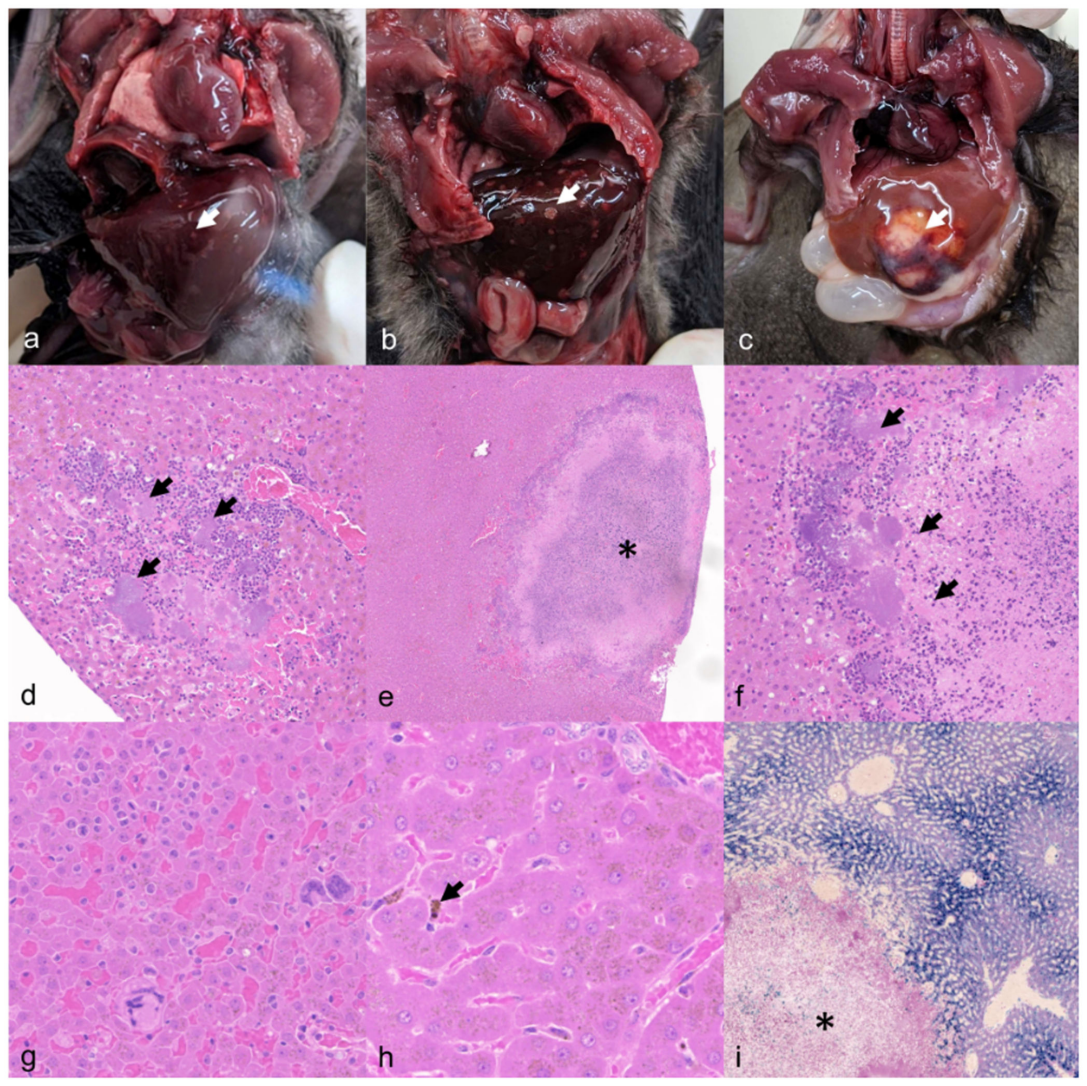
Gross and microscopic lesions of *Yersinia pseudotuberculosis* (Yptb) infection in the liver of Seba’s short-tailed bats (*Carollia perspicillata*). **a**) Early lesions comprise multifocal, faint white/tan discolorations (arrow). Case 1. **b**) Subacute lesions comprise larger, multifocal white/tan discolorations (arrow). Case 7. **c**) Chronic lesions comprise large, white/tan abscess (arrow) with hemorrhagic border. Case 43. **d**) An early lesion comprises of large colonies of coccobacilli (arrows) surrounded by neutrophils, replacing the periportal parenchyma. Case 7. Hematoxylin and eosin (HE). **e**) More advanced lesions comprise focally extensive lytic necrosis (*). Case 7. HE. **f**) Large colonies of coccobacilli (arrows) are present at the edges of the necrotic foci. Case 7. HE. **g**) Away from areas of necrosis, extramedullary hematopoietic cells are commonly present in sinusoids. Case 40. HE. **h**) Hepatocytes and Kupffer cells (arrow) commonly contain speckled brown pigment (hemosiderin) in both Yptb^+^ and, as shown here, Yptb^-^ animals. Case 125. HE. **i**) Hemosiderin is present in all zones of the liver lobule but is most concentrated in periportal hepatocytes, and is largely absent from necrotic foci (*). Case 9. Perl’s Prussian blue.

As with all mortalities at ZSL, all animals received a full gross PME, except for 12 animals that were too autolyzed or mummified; this included carcasses found within water features and animals that had remained in the roosting position after death. Of the 39 confirmed Yptb^+^ animals, changes were primarily in the liver, and presented as multifocal 1-5 mm pink, tan or cream/white discolorations (19/39, Fig. 2a-b), multifocal or focally extensive 5-15 mm cream/white nodules (11/39, Fig. 2c) or both (2/39). Hepatomegaly was subjectively very common but was not reliably recorded in PME notes and so figures are not reported. The next most common gross lesions were splenomegaly (23/39), mesenteric lymphadenomegaly (9/39), multifocal or diffuse reddening of the lungs (7/39) and multifocal reddening of the small intestine and/or red/black intestinal content (5/39). Five of the 39 examined Yptb^+^ animals had no gross lesions.

Histopathology of a wide range of organs (lung, liver, heart, kidney, spleen, brain and intestines) was performed on two animals. Liver lesions comprised large, random areas of lytic necrosis surrounded by few neutrophils and macrophages and large colonies of gram-negative coccobacilli (Fig. 2d-f), while spleens were expanded by large amounts of extramedullary haematopoiesis (EMH). Additionally, there was severe transmural enterocolitis and mild interstitial pneumonia, both characterized by neutrophils and macrophages with large colonies of gram-negative coccobacilli; similar bacteria were commonly observed inside vessels in multiple organs. Liver histology was performed on a further 31 Yptb^+^ cases. Across all 33 livers investigated, the most common finding was intra-sinusoidal EMH (27/33, Fig 2g). Inflammation was multifocal, random and largely pyogranulomatous (11/33) or suppurative (9/33), though in the former neutrophils still predominated. Lytic necrosis was only seen in 16/33 cases and the characteristic large colonies of coccobacilli were only seen in 13/33 cases. In 13/33 cases, EMH was the only finding, but this may be because necrotic areas were out of section as 8/13 had cream/white, tan or pink spotting visible on gross examination.

Yellow/brown stippled, Perl’s Prussian blue positive, cytoplasmic pigment (hemosiderin) was present in hepatocytes and Kupffer cells in 32/33 cases (Fig. 2g-h); this was most concentrated in periportal areas but was found in all parts of the lobule. Minimal Perl’s staining occurred within areas of necrosis, even when there was heavy staining of surrounding liver (Fig. 2i). There was no positive spatial association between the pigment and yersiniosis lesions, suggesting that this was a separate lesion, most likely dietary haemosiderosis (iron overload, iron storage disease), as has been reported in other captive frugivorous bat species. To investigate any association between haemosiderosis and yersiniosis, Perl’s-stained sections of liver were prepared from 27 ME, microbiology negative adult bats, all of which had stainable iron, indicating that the haemosiderosis was not a lesion of yersiniosis. The intensity of Perl’s staining varied widely between bats, from being easily visible at the lowest magnifications to requiring searching at high power.

### Relationship between yersiniosis and hemosiderosis

To determine if there was a relationship between hemosiderosis and susceptibility to yersiniosis, ICP-MS was used to calculate the total elemental iron content of digested samples of frozen liver tissue from the same 27 Yptb^-^ bats and 22 Yptb^+^ bats where frozen tissue was available (Fig. 4a). This showed mean iron concentrations of 5197 µg/g (95% CI 2693-7701 µg/g) in Yptb^-^ bats and 8899 µg/g (95% CI 5247-12551 µg/g) in Yptb^+^ bats, indicating 1.7x as much hepatic iron in the infected population (p=0.0067).

**Figure 3:**
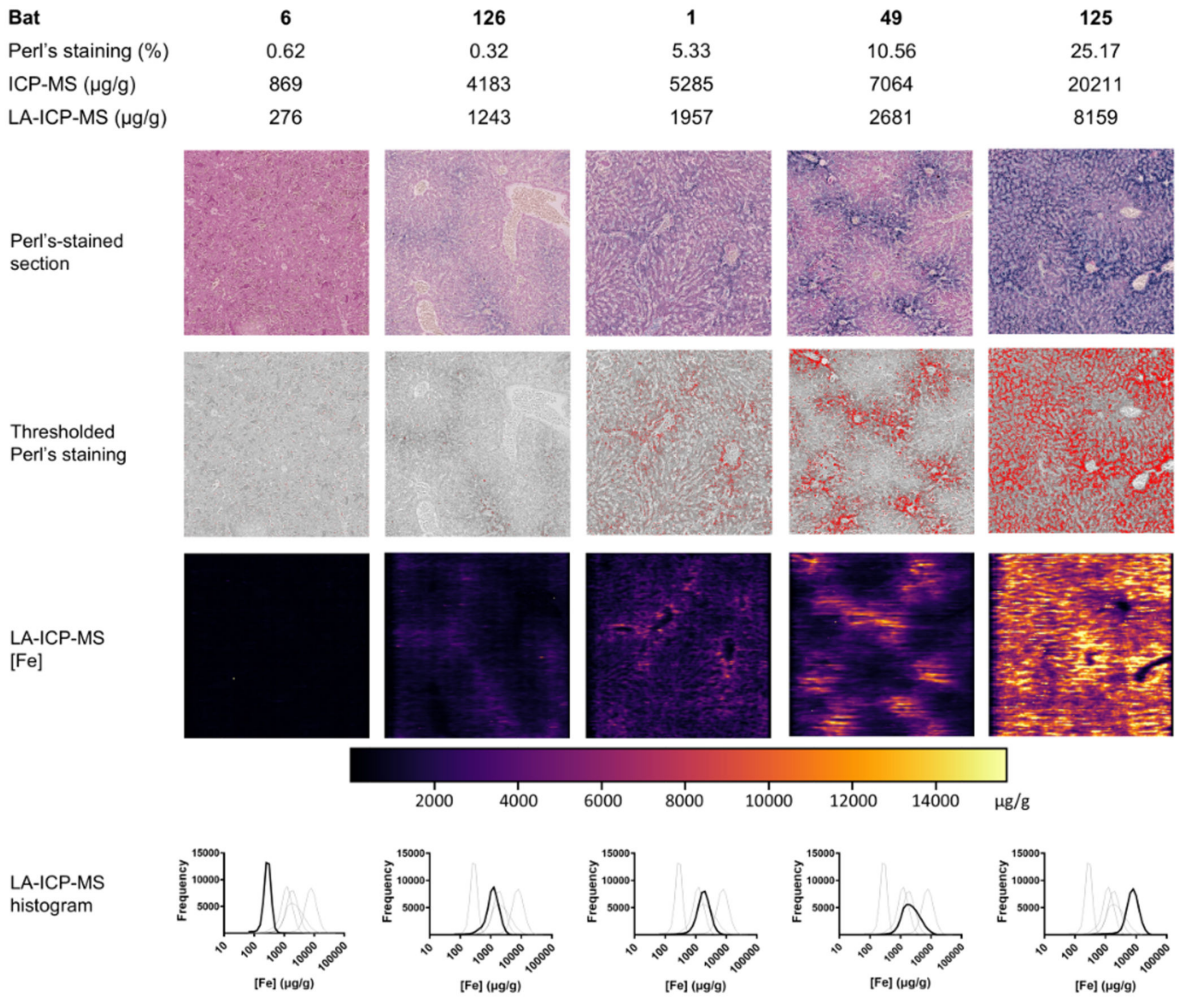
Representative values, images and histograms of three methods of iron quantification in the livers of Seba’s short-tailed bats (*Carollia perspicillata*). Data from five bats with representative levels of hepatic hemosiderosis are shown. Hepatic iron was quantified by three methods: image analysis of Perl’s Prussian blue staining, expressed as a percentage of a 1 mm^2^ regions of interest (ROI) in which blue pixels are identified by color thresholding; ICP-MS, in which frozen tissue is digested and iron quantified by inductively-coupled mass spectrometry; and LA-ICP-MS, in which laser-ablation is used to vaporize voxels of tissue (in the same ROI from a sequential section of tissue) and quantify iron concentrations by ICP-MS at a 5 µm resolution. LA-ICP-MS data are expressed as a heatmap (“LA-ICP-MS [Fe]”), histogram (“LA-ICP-MS histogram”) or as the mean concentration across all 160,000 voxels in the ROI (“LA-ICP-MS, (µg/g)”).

Two further techniques were then used to independently quantify the degree of hemosiderosis in each liver and to investigate its spatial distribution. Firstly, the “analyze particles” function of ImageJ was used to calculate the percentage of a 1 mm^2^ ROI that was positively stained by Perl’s Prussian blue (image analysis, IA); this was performed on three ROI each from single sections from 27 Yptb^-^ and 24 Yptb^+^ bat livers. Secondly, LA-ICP-MS was used to image the distribution of iron quantitatively in of each individual 5 x 5 x 4 µm (100 µm^3^) voxel in consecutive sections of the same FFPE tissue blocks used for IA, allowing the same ROI to be used. Due to available resources, this technique was performed on sections from 10 Yptb^-^ and 10 Yptb^+^ bat livers. These data were expressed as heatmaps, as the mean iron concentration across the whole ROI and as histograms of the frequency each concentration across the ROI (Fig. 3).

**Figure 4:**
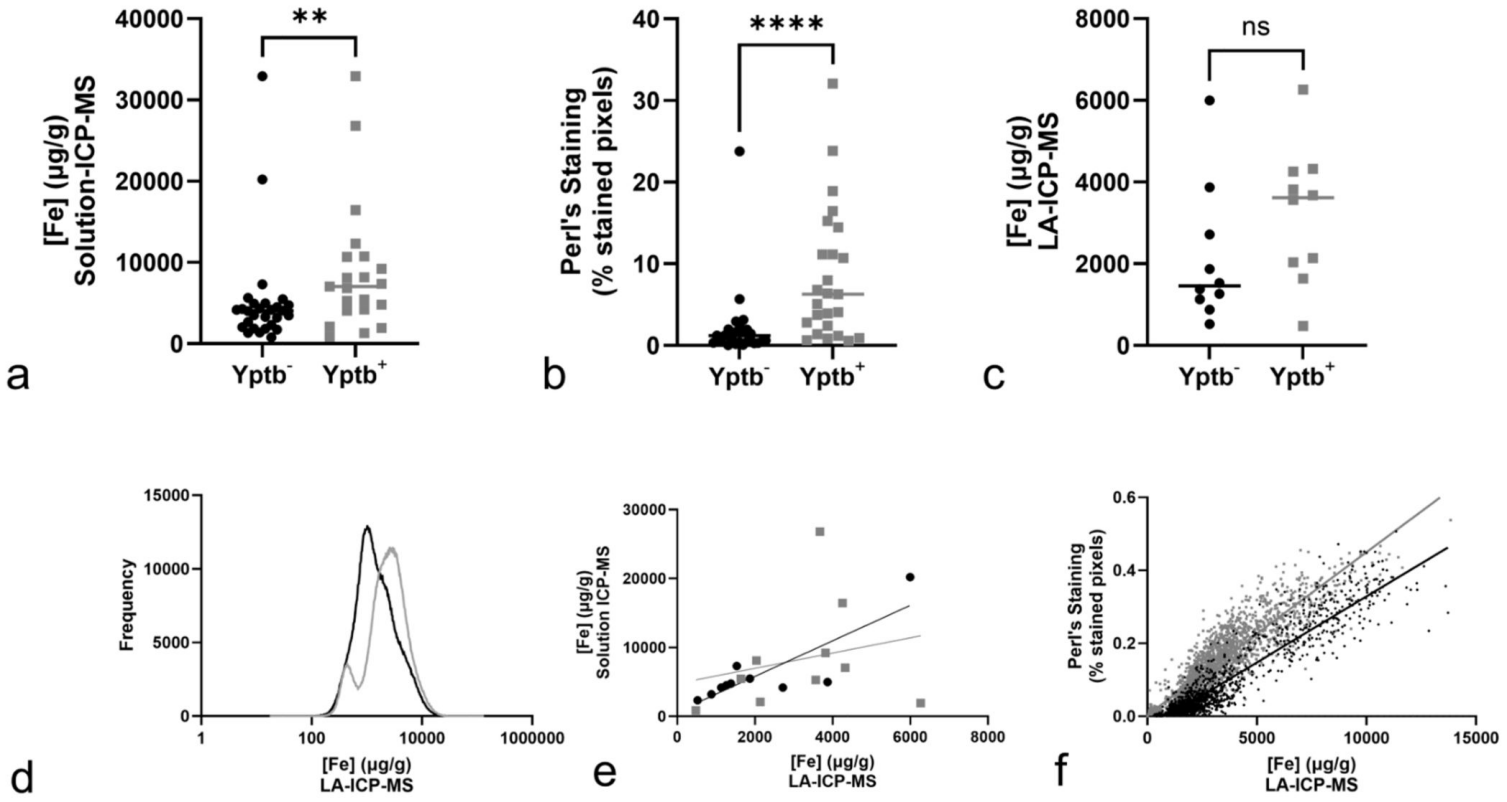
The relationship between hepatic iron concentrations and infection by *Yersinia pseudotuberculosis* (Yptb) in Seba’s short-tailed bats (*Carollia perspicillata*). Iron was quantified by solution inductively-couple mass spectrometry (ICP-MS) (**a**), image analysis of Perl’s stained sections (**b**), and laser-ablation (LA) ICP-MS (**c**). The LA-ICP-MS data can also be expressed as frequency distributions of all 100 µm^3^ voxels (n= 1031400) in each group (**d**). Non-linear regression is used to compare solution ICP-MS (**e**) and image analysis (**f**) to LA-ICP-MS. In all cases black circles and lines indicate Yptb^-^ animals and grey squares and lines Yptb^+^ animals. Horizonal lines indicate the median value and individual points represent the mean of three technical replicates, except for the solution ICP-MS which was performed in singlicate, and in (f) where each point represents a single 0.01 mm^2^ area of tissue. Pairwise comparisons indicate p values from two-tailed Mann-Whitney tests. **** indicates p < 0.0001; **, p < 0.01; ns, not significant.

IA (Fig. 4b) concurred with solution ICP-MS, with Yptb^+^ animals having substantially higher levels of Perl’s staining (p < 0.0001). Perl’s staining occupied a mean of 2.16% (95% CI 0.38-3.93%) of the area of each ROI from Yptb^-^ bats, but was nearly 4x more abundant at 8.37% (95% CI 5.03-11.7%) in Yptb^+^ bats.

LA-ICP-MS (Fig. 4c) did not show a statistically significant relationship (p = 0.1051) between hepatic iron and yersiniosis. However, the mean iron concentrations of 2116 µg/g (95% CI 919.3-3313 µg/g) for Yptb^-^ bats and 3219 µg/g (95% CI 2031-4407 µg/g) for Yptb^+^ bats indicate 1.5x as much hepatic iron in the infected than uninfected population, a very similar ratio to that measured with solution ICP-MS. This suggests that the lack of significance may be due to the lower number of samples available for LA-ICP-MS and a Type II statistical error.

LA-ICP-MS combines the quantitative ability of solution ICP-MS while maintaining the spatial information provided by Perl’s staining. LA-ICP-MS showed that the total iron (ferric and ferrous) distribution correlated well with hemosiderin (ferric only) staining (Fig. 3), indicating that the ferrous iron was largely insignificant and that Perl’s staining provided a reasonable approximation of total liver iron.

It is possible that local concentrations of iron are more important than average hepatic iron concentration; when multiple bacteria embolize into a tissue, those that settle in areas of high iron concentration may be more successful at colonization than those that settle in low iron areas. Iron concentrations in Seba’s bat livers are highly heterogeneous. In case 125 the mean iron concentration by LA-ICP-MS of all three ROI was 7736 µg/g but the standard deviation was 4016 µg/g, with the bottom 1% of voxels below 4 µg/g and the highest 1% in excess of 20,295 µg/g. Thus, it may be that a liver with low average iron, but a few “hotspots” of very high iron concentrations is more susceptible to bacterial infections than one with diffusely moderate iron concentrations.

To investigate this, frequency distributions of voxels by iron concentration were constructed for each individual (examples in Fig. 3) and for both populations (Fig. 4d). An increase in total liver iron correlated with a right-shift in the frequency distribution of iron concentration per 100 µm^3^ voxel.

In the bats with the lowest mean hepatic iron, there is approximately 2.8x more iron in the highest (90^th^ percentile) areas than the lowest (10^th^ percentile); in the bats with the most iron, an 11x increase in average hepatic iron translates into just a 1.5x increase in this ratio. This right-shift without a change in the distribution shape indicates that, while the periportal bias is maintained, the increase in iron storage is even across the lobule, contradicting the “hotspot” hypothesis.

The three methods were compared against each other using non-linear regression. Solution ICP-MS correlated well with LA-ICP-MS in Yptb^-^ livers, with a linear fit through the origin and an R^2^ of 0.71; however, there was no correlation in Yptb^+^ livers (R^2^ = 0.054, Fig. 4e). This discrepancy is most likely due to the presence of Yptb^+^ lesions. These lesions are very low in iron and show minimal or absent Perl’s staining; LA-ICP-MS of a necrotic lesion in case 20 showed an average iron concentration of just 272 µg/g in an ROI composed solely of lytic necrosis compared to 2080 µg/g in viable tissue. As frozen liver had been collected opportunistically before this study was designed, there was no consistency in whether the tissue did or did not contain areas of necrosis or abscessation, potentially explaining the poor correlation.

As the IA and LA-ICP-MS were performed on near-identical serial sections, ROI from both Yptb^+^ and Yptb^-^ sections could be compared together without any concerns about heterogeneity, as necrotic, inflamed and autolyzed areas could easily be excluded. To increase the accuracy of the regression, each 1 mm^2^ ROI was subdivided into 100 smaller 0.01 mm^2^ ROIs and IA and LA-ICP-MS values were compared (Fig. 4f). There was a linear relationship between IA and LA-ICP-MS values in both Yptb^-^ (R^2^ = 0.895) and Yptb^+^ (R^2^ = 0.837) bats. However, the two data sets did not share the same linear function (p < 0.0001, F = 2042, extra sum-of-squares F test), with the Yptb^+^ population generally showing greater Perl’s staining. The x intercept for the Yptb^-^ curve is 910 µg/g iron, suggesting that Perl’s staining does not occur below that concentration; this is consistent with the fact that Perl’s staining will only be visible above a certain threshold and demonstrates the greater sensitivity of LA-ICP-MS to Perl’s staining.

### Hemosiderosis and pregnancy

To investigate whether iron was responsible for the increased susceptibility of non-pregnant females, the relationship between sex, pregnancy status and hepatic iron was examined in Yptb^-^ bats. Only solution ICP-MS data were examined, as there was no LA-ICP-MS data for male bats or gravid female bats. Mean hepatic iron was 4489 µg/g (95% CI 2868-6111 µg/g) in female bats, 1.92x higher than the mean for male bats 2335 µg/g (95% CI 496.9-4172 µg/g); this difference was not statistically significant (p=0.1262) likely due to the very low number of male bats available for study due to sex matching to the Yptb^+^ population, where males were under-represented. Among female bats, the mean hepatic iron of pregnant animals was 2.14x higher (6649 µg/g, 95% CI 2645-10653 µg/g) than in non-pregnant animals (3101 µg/g, 95% CI 2329-3874 µg/g), which was significant (p=0.0027).

## Discussion

This study is, to the best of the authors’ knowledge, the first to investigate an association between hemosiderosis and yersiniosis in a non-experimental, epidemic scenario. It is also the first study to compare high-resolution spatial mass spectrometry (LA-ICP-MS) with a quantitative assessment of Perl’s staining (IA).

Epizootics of Yptb in closed colonies of bats in zoological collections have been previously reported, including an outbreak in 3/400 (1%) Seba’s short-tailed bats^32^ and 80/115 (70%) and 12/61 (20%) of two colonies of Egyptian fruit bats.^17,46^ The outbreak described in this study affected 51/222 (23%) adult Seba’s short-tailed bats, contradicting the hypothesis of Hahn et al. that Seba’s bats may be more resistant to Yptb than Egyptian fruit bats. The outbreak described by Hahn et al. is too small for statistical analysis, but the affected animals comprised two females (one pregnant, one recently aborted) and one male. Childs-Sanford et al. report no statistically significant association between sex and Yptb infection while Nakamura et al. do not provide any signalment data; neither study makes mention of pregnancy.

In contrast, in this study, females significantly outnumbered males in the Yptb^+^ cohort (odds ratio 3.4) and pregnant females were substantially more likely to be in the Yptb^-^ cohort than non-pregnant females (odds ratio 13.6). It is possible that fewer pregnant animals were present in the Yptb^+^ cohort because they had aborted but, if so, this must have occurred in the early stages of pregnancy as the animals did not have enlarged uteri or mammary glands. The enclosure was regularly cleaned and inspected, and no aborted fetuses were discovered during or in the three weeks before the outbreak.

The disproportionate representation of females, but not pregnant females, in the Yptb^+^ population stands in contrast to many studies in humans and animal models showing that males are more susceptible than females to bacterial infections, including *Y. enterocolitica*.^41,60,73^ While this difference is attributed to immunological, genetic and hormonal factors, there is a significant role for behavioral differences as well, which may account for the differences seen here. The sex ratio in the total population was 83:134 male:female, a ratio of 1.6, which is consistent with the ratio of 1.57 ± 0.37 (SD) reported in wild caught Seba’s bats in the wet season of the Brazilian Amazon.^57^ However, in the wild, the local sex ratio can vary depending on the type of habitat and whether or not it is the breeding season, possibly due to the need for a greater positive energy balance for females during, or in preparation for, pregnancy.^57^ Pregnant Seba’s bats fly shorter distances to forage than males and non-pregnant females^16^ and so may congregate closer to feeding stations; this could lead to segregation of the pregnant population, protecting them from transmission of the infection. Similarly, there is likely greater interaction between males and non-pregnant females than there is with pregnant females, due to mating behaviors, which may further protect pregnant females from being exposed. The lack of sex or pregnancy effects in other reported epizootics may be due to differences in husbandry, such as differences in the distribution of feeding stations, or due to breeding differences. There was no seasonal variation in this population so breeding occurred year-round; in enclosures with seasonal variation outbreaks may simply have occurred outside of the breeding season.

The major gross and histopathological findings of multifocal, suppurative to pyogranulomatous hepatitis, lymphadenitis and splenitis were consistent with previous reports,^17,32,46^ though this study was the only one to report hepatic EMH. EMH was the most common histological finding in this study and was unique to the Yptb^+^ cohort; this may be caused by increased demand for leukocytes induced by the extensive inflammation or may be directly induced by the bacteria, as *Yersinia enterocolitica* is known to induce the production of pro-myelopoietic cytokines.^35^

Although not caused by Yptb infection, hepatic hemosiderosis was the most common lesion, occurring in 59/60 (98%) livers from both infected and uninfected bats. Hemosiderosis (iron overload, or iron storage disease) is well described in a variety of frugivorous bat species, including Seba’s bats.^18,20,24,36^ Perl’s Prussian blue staining was performed on sections of liver from museum specimens of five wild-caught Seba’s short-tailed bats and no staining was observed (Karen Sears, personal communication), suggesting that hemosiderosis is likely the result of excessive dietary iron in captive bats rather than an intrinsic feature of the species.

Despite the high prevalence of hemosiderosis (iron overload without associated hepatoxicity), no cases of hemochromatosis (iron overload with associated hepatoxicity) were identified, in contrast to previous studies which have described necrosis and bridging fibrosis (cirrhosis).^36^ The same study also showed that bats with hemochromatosis were significantly more likely to have hepatocellular carcinoma than those with hemosiderosis. No incidences of neoplasia were detected in this study, though gross examination of bats euthanized during depopulation was limited. In humans, hepatocellular carcinoma is associated with increased hepatic iron, though it is not definitively proven that the latter causes the former. ^22,23,45^ The proposed mechanism for carcinogenesis by iron storage is oxidative stress, the same mechanism by which it is cytotoxic;^71^ thus the sub-toxic levels of iron in these livers may have been insufficient to cause carcinogenesis.

**Figure 5:**
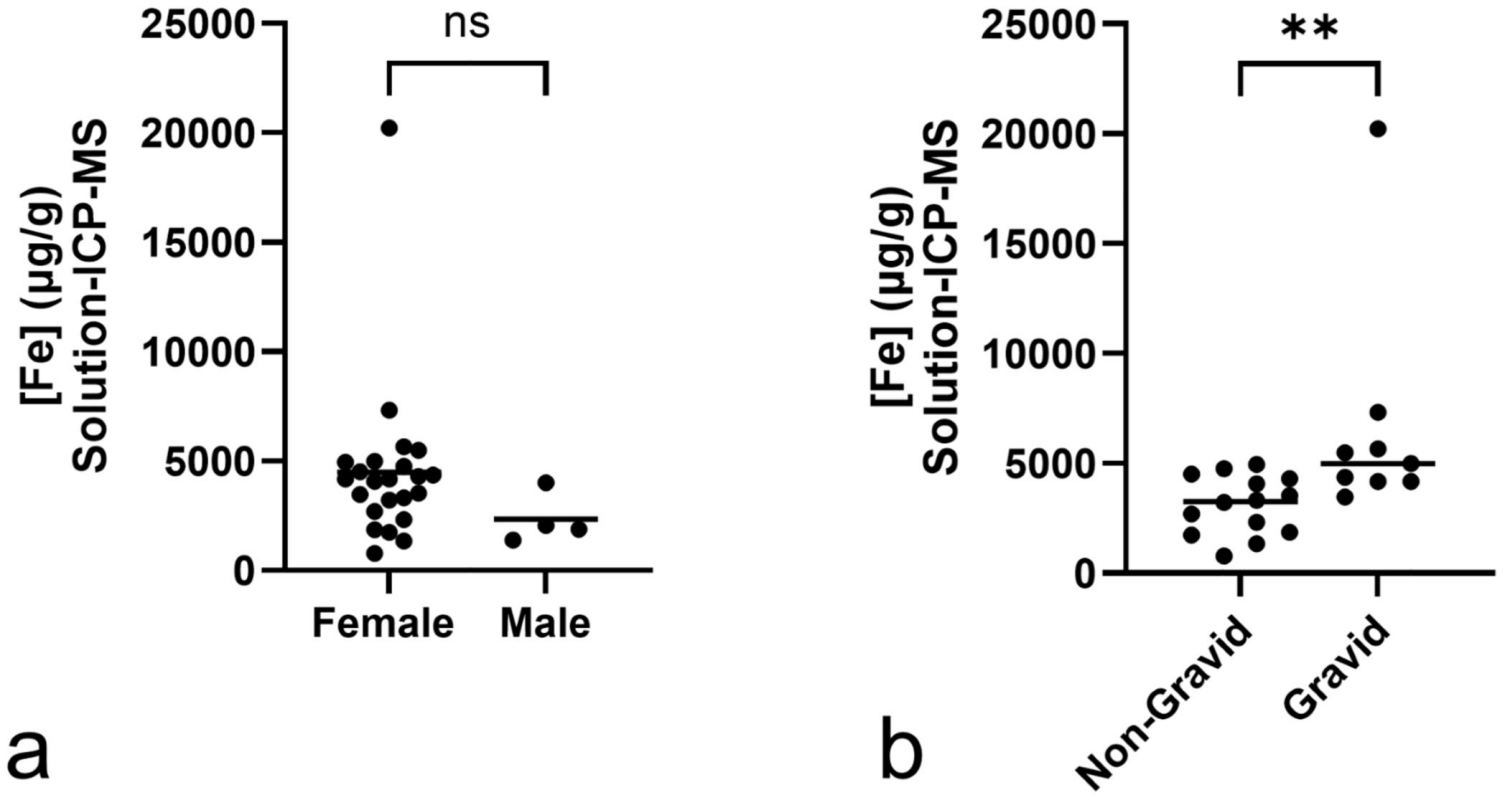
Hepatic iron concentrations in male and female (**a**) and gravid and non-gravid female (**b**) Seba’s short-tailed bats (*Carollia perspicillata*). Data are in singlicate and represent 23 female (9 of which gravid) and 4 male bats. ** indicates p < 0.01; ns, not significant.

Hepatic iron concentrations were higher, on average, in female bats than male bats, though this was not statistically significant (Fig. 5a). However, increased hepatic iron alone cannot explain the sex difference, as gravid bats had significantly higher concentrations than non-gravid bats (Fig. 5b), despite the latter being substantially over-represented in the infected population. In humans, fetal development requires iron so pregnancy commonly results in iron-deficiency in the mother,^11^ thus it is surprising to see a negative correlation between hepatic iron concentration and pregnancy status. This is also in contrast to studies in Sumatran rhinoceros (*Dicerorhinus sumatrensis*), where pregnancy is correlated with a progressive decrease in serum ferritin, and it is thought that pregnancy is a useful management strategy to prevent hemochromatosis in this species.^61^ This discrepancy may be due to increased feeding on iron-replete feed during pregnancy combined with decreased menstruation.^55^

We hypothesized that animals with higher concentrations of hepatic iron would be more susceptible to systemic Yptb infection than those with lower levels. As this is a descriptive study, there is no defined control group of animals that can be said to have “normal” levels of iron. Of the livers that received LA-ICP-MS, only 1/20, case 6, had no Perl’s staining. The average iron concentrations in this liver by LA-ICP-MS was 494 µg/g; the lowest average iron concentration in a liver that had visible Perl’s staining was 666 ± 48 µg/g (case 341). This suggests that bat livers become Perl’s positive when average iron concentrations exceed somewhere between 494 and 666 µg/g. However, the highest non-staining ROI from case 6 was 602 µg/g and the lowest from case 114, which did have visible Perl’s staining, was 612 µg/g. While it is possible that liver sections suddenly become Perl’s positive in this narrow window, it is likely that there are confounding factors involved, such as the presence of non-stainable ferrous (Fe^2+^) iron and/or heterogeneity in iron distribution.

To investigate the hypothesis, we thus had to compare infection to the degree of hemosiderosis, rather than its presence or absence. All three methods of quantification showed greater levels of hepatic iron in the infected population than the uninfected population (Fig. 4a-d), though the LA-ICP-MS data were not statistically significant, possibly due to the lower sample size.

Quantification of hepatic iron by solution ICP-MS produced data that correlated well with alternative methods in Yptb^-^ animals, but less well in Yptb^+^ animals. This may be due to the presence of areas of inflammation and/or necrosis that were depleted in iron. Where this iron goes is not clear; while some is undoubtedly taken up into the proliferating bacilli, LA-ICP-MS did not show high concentrations of iron within bacterial colonies, and certainly not enough to account for all that had been lost. It is most likely that lysis of the hepatocytes released the hemosiderin into blood, lymph or bile, whereupon it was removed from the liver; this solubilized iron may even leave the body into the damaged intestine if there is concurrent *Yersinia* enteritis.

To avoid this problem, spatially selective techniques that could be targeted to areas without *Yersinia* were employed. Perl’s staining is the standard method for qualitatively evaluating the level of hemosiderosis in tissue sections; this was supplemented with image analysis. Image analysis can only be semi-quantitative as it is only measuring the percentage of the section that is stained by the Perl’s reagent, not the intensity of the staining, and it only measures ferric (Fe^3+^) iron. To quantitatively measure the iron content of each section, LA-ICP-MS was employed, which combines the sensitivity of mass spectrometry with the spatial mapping of histology at a 5 µm resolution.

There was a strong linear relationship between iron concentrations estimated by Perl’s staining and measured by LA-ICP-MS (Fig. 4f), as previously described between Perl’s staining and biochemical iron quantification methods,^51^ confirming that this cheap and readily available method provides a reasonable approximation of true hepatic iron. However, different correlations were obtained for Yptb^+^ and Yptb^-^ tissues, with Yptb^+^ sections staining slightly more strongly with Perl’s stain than those from Yptb^-^ bats.

This artifact is likely best explained by autolysis of the tissue. All Yptb^-^ tissues were obtained during depopulation ME, with carcasses immediately chilled and tissues fixed or frozen within 3 hours of death. Conversely, most Yptb^+^ tissues came from bats that were found dead, and so may have sat at ambient temperatures for hours or days before chilling. As the first week of the outbreak corresponded with the start of the first national covid-19 lockdown in the UK, the earliest carcasses then waited up to 10 days (median 2 days) for PME. Autolysis could produce dark staining background artifacts that were mistaken for Perl’s staining by the IA algorithm. It is also possible that autolysis could truly have decreased hepatic iron concentrations, such as by leaching of soluble iron into surrounding tissues, but this is unlikely as liquid storage of liver tissue in formalin does not substantially affect concentrations over time, suggesting little hepatic iron is readily diffusible.^13^ Prolonged storage in formalin may also result in acid hematin (“formalin pigment”) that, although Perl’s negative, is pigmented and so may be mislabeled as hemosiderin by the image analysis algorithm; however, no acid hematin was observed in any of the sections.

For these reasons, LA-ICP-MS may be a more appropriate method for determining hepatic iron than ICP-MS, the routine method, when there is extensive necrosis in a sample. Image analysis of Perl’s staining is a cheap alternative to LA-ICP-MS, but is only semi-quantitative and may not be appropriate in cases where there is autolysis.

Another significant limitation in the study is the lack of age data. The overrepresentation of non-pregnant females in the Yptb^+^ population could be due to increased infection rates among older, post reproductive animals. Similarly, one would reasonably expect hemosiderosis to positively correlate with age, so older animals may be more at risk of both hemosiderosis and yersiniosis without any direct link between the two conditions. The only way to control for these confounding factors would be by performing carefully controlled experimental infections. Such experiments need not be performed on mammals; many strains of Yptb are pathogenic in the popular nematode model *Caenorhabditis elegans*,^34^ for which iron overload models are well described.^59^ However, a pilot experiment demonstrated that the Yptb strain from this specific outbreak is not pathogenic to *C. elegans* (Jonathan Hodgkin, personal communication).

In conclusion, we report an outbreak of Yptb in captive Seba’s short-tailed bats, describing the gross and histological features of the infection. Hemosiderosis was widespread in the population and infected bats had an average of 1.4 and 1.7x higher hepatic iron concentrations than uninfected bats, as determined by LA-ICP-MS and ICP-MS respectively. Contrary to previous studies, non-pregnant females were overrepresented in the infected population, possibly due to behavioral segregation; this could not be explained by increased hepatic iron. We hypothesize that increased hepatic iron storage makes animals more susceptible to systemic Yptb infection as it limits the efficacy of hepcidin induced iron sequestration,^48^ leaving more iron available for the bacteria to metabolize. To our knowledge, this is the first evidence for an association between iron storage and Yptb in an epidemic scenario.

## Supplementary Material

Supplementary Materials
