# Supplementary material for: Hemosiderosis is associated with increased susceptibility to Yersinia pseudotuberculosis infection in Seba’s short-tailed bats (Carollia perspicillata)
Source: Vet Pathol. Author manuscript; Available in PMC 2025 Nov 13. (PMC7618344; doi:10.1177/03009858251343017)
Supplement: Supplementary Materials [file EMS210402-supplement-Supplementary_Materials.pdf]

Supplemental Materials

Hemosiderosis is associated with increased susceptibility to *Yersinia pseudotuberculosis* infection in Seba's short-tailed bats (*Carollia perspicillata*)

Simon Spiro, Alexander Griffiths, Ahmad Arnaout, Ethan Wrigglesworth, Shaheed Karl Macgregor, Shinto Kunjamma John, Stamatios Alan Tahas, Emma Nye, Alexander P. Morrell

Supplemental Table S1. Experimental parameters and data acquisition parameters used for laser-ablation inductively-couple mass spectrometry (LA-ICP-MS) imaging.

	Bat livers	NIST 612 Scans	Gels
**Teledyne Photon Machines Iridia**			
Energy density (J cm^{-2})	0.5	3	0.5
Repetition rate (Hz)	1000	100	1000
Scan speed (µm s^{-1})			
Beam waist diameter (µm)	5 (circle)	35 (square)	5 (circle)
Scanning mode	Fixed Dosage	Fixed Dosage	Fixed Dosage
Scanning direction	Uni-directional	Uni-directional	Uni-directional
Effective dosage (shots per position)	10	10	40
Helium carrier gas flow rate (L min^{-1})	0.3	0.3	0.3
Washout (ms)	20	20	20
**Thermo Fisher Scientific iCAP TQ ICP-MS**			
RF power (W)	1550	1550	1550
Ar plasma gas flow rate (L min^{-1})	14	14	14
Ar auxiliary gas flow rate (L min^{-1})	0.8	0.8	0.8
Nebuliser gas flow rate (L min^{-1})	1.03	1.03	1.03
CR gas flow rate (L min^{-1})	0.16 (O_2)	0.16 (O_2)	0.16 (O_2)
ICP-MS mode	TQ	TQ	TQ
Acquired m/z ratios (amu)	^{56}Fe	^{56}Fe	^{56}Fe
Respective dwell times (ms)	10	10	10
Total scan cycle time (ms)	10	10	10

Supplemental Table S2. Details of the 222 Seba's short-tailed bats (*Carollia perspicillata*) in the study population. Pregnancy status was subjectively categorized as early if only membranes or an indistinct fetus was observed, mid if there was a recognizable but non-viable fetus, or late if there was a large, near-term fetus. Abbreviations: NR, not recorded; F, female; M, male; ME, management euthanasia; U, undiagnosed; E, excluded.

Case	Date of death	Weight (g)	Sex	Manner of death	Elbow-carpus length (mm)	Pregnancy status	Alopecia	Yersinia status
**1**	14/03/20	19.5	F	Died	40		NR	+
**2**	21/03/20	26	M	Died	44		Marked	U
**3**	14/03/20	10.3	M	Died	38		NR	+
**4**	16/03/20	6.8	F	Died	36		NR	U
**5**	16/03/20	NR	F	Died	42		NR	E
**6**	19/03/20	15.9	F	Died	NR		Mild	+
**7**	22/03/20	17	F	Died	40		Marked	+
**8**	13/03/20	16.6	F	Died	40		Moderate	U
**9**	23/03/20	20	F	Died	42		NR	+
**10**	23/03/20	15	F	Died	43		NR	+
**11**	25/03/20	15.9	F	Died	40		Moderate	+
**12**	25/03/20	17.6	F	Died	43		Severe	+
**13**	26/03/20	13.2	F	Died	39		Mild	+
**14**	26/03/20	17.5	M	Euth	41.5		Mild	+
**15**	30/03/20	16.5	F	Died	42.5		NR	U
**16**	31/03/20	17.6	M	Died	41		NR	U
**17**	31/03/20	17.5	F	Euth	40		NR	+
**18**	04/04/20	17.6	F	Died	41		Mild	+
**19**	05/04/20	15.5	F	Died	41		Marked	+
**20**	04/04/20	16.1	F	Died	41		Mild	+
**21**	04/04/20	NR	NR	Died	NR		NR	U
**22**	04/04/20	NR	NR	Died	NR		NR	U
**23**	04/04/20	NR	NR	Died	NR		NR	U
**24**	04/04/20	NR	NR	Died	NR		NR	U
**25**	04/04/20	NR	NR	Died	NR		NR	U
**26**	07/04/20	17	F	Euth	41		Moderate	+
**27**	09/04/20	17.7	M	Died	41		Mild	+
**28**	12/04/20	20.4	F	Died	42		Marked	+
**29**	13/04/20	15.8	F	Died	39		Moderate	+
**30**	14/04/20	20.5	F	Died	43		Mild	+
**31**	19/04/20	NR	M	Died	NR		NR	U
**32**	14/04/20	16.6	F	Died	39		Mild	+
**33**	18/04/20	20.4	F	Died	43		NR	+
**34**	18/04/20	16.5	F	Died	40		NR	+
**35**	18/04/20	17	M	Died	42		Moderate	+
**36**	23/04/20	17.8	F	Died	40		NR	+
**37**	28/04/20	23.2	F	Died	42		NR	+

**38**	01/05/20	14.9	M	Died	42		Mild	+
**39**	02/05/20	15.6	F	Died	40		Moderate	+
**40**	03/05/20	18.7	F	Died	43		Severe	+
**41**	05/05/20	19.1	F	Died	41		Mild	+
**42**	06/05/20	19.4	M	Died	40		Marked	+
**43**	07/05/20	21.4	F	Died	41	Early	Marked	+
**44**	12/05/20	16.5	F	Died	42		Marked	+
**45**	14/05/20	24.5	F	Died	41	Early	Severe	+
**46**	15/05/20	18.5	F	Died	40		Marked	+
**47**	19/05/20	19.1	F	ME	41		Severe	+
**48**	22/05/20	16.8	F	ME	41		Mild	+
**49**	22/05/20	18	F	ME	41		None	+
**50**	26/05/20	20	F	ME	42		Mild	+
**51**	30/05/20	19	M	Died	39.5		Marked	+
**52**	26/03/20	23.5	M	Died	41		Marked	E
**53**	20/04/20	17.5	F	ME	NR		Mild	-
**54**	20/04/20	17.8	F	ME	NR		Moderate	-
**55**	20/04/20	18	F	ME	NR		Severe	-
**56**	20/04/20	18	M	ME	NR		Marked	-
**57**	20/04/20	17	M	ME	NR		None	-
**58**	20/04/20	18	M	ME	NR		Severe	-
**59**	20/04/20	18.5	M	ME	NR		None	-
**60**	20/04/20	19.1	M	ME	NR		Severe	-
**61**	20/04/20	17	M	ME	NR		Marked	-
**62**	20/04/20	16.6	M	ME	NR		Moderate	-
**63**	20/04/20	18.6	F	ME	NR		Moderate	-
**64**	20/04/20	18.9	M	ME	NR		Severe	-
**65**	20/04/20	19.1	M	ME	NR		None	-
**66**	20/04/20	18	M	ME	NR		Moderate	-
**67**	20/04/20	16.9	F	ME	NR		Mild	-
**68**	20/04/20	16.8	F	ME	NR		Mild	-
**69**	20/04/20	16.8	F	ME	NR		Moderate	-
**70**	20/04/20	16	F	ME	NR		Mild	-
**71**	20/04/20	20	M	ME	NR		Marked	-
**72**	20/04/20	19	M	ME	NR		Marked	-
**73**	20/04/20	17.5	F	ME	NR		Mild	-
**74**	20/04/20	14.4	M	ME	NR		Moderate	-
**75**	20/04/20	18.3	M	ME	NR		Moderate	-
**76**	20/04/20	19.4	M	ME	NR		Marked	-
**77**	20/04/20	14.5	M	ME	NR		Moderate	-
**78**	20/04/20	15.2	F	ME	NR		Mild	-
**79**	19/05/20	19.1	M	ME	43		Marked	-
**80**	19/05/20	17.8	F	ME	40		None	-
**81**	19/05/20	18.3	M	ME	42		Marked	-
**82**	19/05/20	20.5	F	ME	42	Mid	Moderate	-
**83**	19/05/20	20	M	ME	45		Marked	-
**84**	19/05/20	24.5	F	ME	43		Mild	-

**85**	19/05/20	19.7	M	ME	42		Severe	-
**86**	19/05/20	16.7	F	ME	40		Severe	-
**87**	19/05/20	20	M	ME	42		Severe	-
**88**	19/05/20	21.9	F	ME	43		Moderate	-
**89**	19/05/20	21.6	M	ME	43		Severe	-
**90**	19/05/20	16.3	F	ME	42		Moderate	-
**91**	19/05/20	18	F	ME	42		None	-
**92**	19/05/20	18.4	F	ME	41	Late	Marked	-
**93**	19/05/20	21.6	F	ME	43	Late	Marked	-
**94**	19/05/20	23.6	F	ME	41	Late	Marked	-
**95**	19/05/20	20	F	ME	43	Mid	Severe	-
**96**	19/05/20	20	F	ME	42	Mid	None	-
**97**	19/05/20	20.9	F	ME	41	Mid	None	-
**98**	19/05/20	16.4	F	ME	42		Mild	-
**99**	19/05/20	20	M	ME	42		Marked	-
**100**	19/05/20	22.6	M	ME	44		Mild	-
**101**	19/05/20	20.7	M	ME	42		None	-
**102**	19/05/20	20.3	M	ME	44		Marked	-
**103**	19/05/20	19.3	M	ME	43		None	-
**104**	19/05/20	18.5	M	ME	42		Severe	-
**105**	19/05/20	17	F	ME	44		None	-
**106**	19/05/20	16.6	F	ME	42		Moderate	-
**107**	19/05/20	17.7	F	ME	41	Early	Moderate	-
**108**	19/05/20	19.6	F	ME	40	Mid	Severe	-
**109**	19/05/20	17.2	F	ME	42		Marked	-
**110**	19/05/20	18.5	F	ME	43		Mild	-
**111**	19/05/20	17.4	F	ME	42		Moderate	-
**112**	19/05/20	15.1	F	ME	40		None	-
**113**	19/05/20	19.4	F	ME	41	Mid	None	-
**114**	19/05/20	17.2	F	ME	43		Mild	-
**115**	19/05/20	15.8	F	ME	41		Marked	-
**116**	19/05/20	17.4	F	ME	41		None	-
**117**	19/05/20	18.8	F	ME	44	Mid	None	-
**118**	19/05/20	17.7	F	ME	44		Severe	-
**119**	19/05/20	19.3	M	ME	42		Moderate	-
**120**	19/05/20	20.8	M	ME	44		Severe	-
**121**	19/05/20	18.3	F	ME	43		Moderate	-
**122**	22/05/20	17.1	M	ME	43		Marked	-
**123**	22/05/20	19.4	M	ME	46		Moderate	-
**124**	22/05/20	18.1	F	ME	41		None	-
**125**	22/05/20	21.5	F	ME	44	Late	Mild	-
**126**	22/05/20	19	F	ME	43	Mid	None	-
**127**	22/05/20	17.2	F	ME	43	Early	None	-
**128**	22/05/20	17.2	F	ME	40	Early	None	-
**129**	22/05/20	17.5	F	ME	43		Marked	-
**130**	22/05/20	25.2	F	ME	45	Late	None	-
**131**	22/05/20	17.5	F	ME	41		Moderate	-

**132**	22/05/20	18.3	F	ME	43	Late	Moderate	-
**133**	22/05/20	19.4	F	ME	41	Mid	Mild	-
**134**	22/05/20	18.5	F	ME	43		Severe	-
**135**	22/05/20	19.2	F	ME	43	Mid	Mild	-
**136**	22/05/20	22	F	ME	43	Late	Moderate	-
**137**	22/05/20	19	F	ME	40	Mid	Moderate	-
**138**	22/05/20	19.7	M	ME	42		Mild	-
**139**	22/05/20	15.2	M	ME	41		Moderate	-
**140**	22/05/20	19.2	M	ME	44		Mild	-
**141**	22/05/20	23.5	M	ME	44		Severe	-
**142**	22/05/20	17.7	M	ME	40		Severe	-
**143**	22/05/20	18	M	ME	45		Mild	-
**144**	22/05/20	20.9	M	ME	41		None	-
**145**	22/05/20	18	M	ME	44		Marked	-
**146**	22/05/20	19	M	ME	44		Marked	-
**147**	22/05/20	19.6	M	ME	42		Marked	-
**148**	22/05/20	20.3	M	ME	42		Mild	-
**149**	22/05/20	20.6	M	ME	44		Moderate	-
**150**	22/05/20	20	M	ME	43		Severe	-
**151**	22/05/20	20.7	M	ME	43		Marked	-
**152**	22/05/20	18.9	M	ME	43		Moderate	-
**153**	22/05/20	17.7	M	ME	44		Mild	-
**154**	26/05/20	17.5	M	ME	42		Severe	-
**155**	26/05/20	20	F	ME	42	Mid	None	-
**156**	26/05/20	19.4	M	ME	41		Mild	-
**157**	26/05/20	19.4	F	ME	39.5	Mid	Marked	-
**158**	26/05/20	18.8	M	ME	44		Mild	-
**159**	26/05/20	20.8	F	ME	40	Mid	Mild	-
**160**	26/05/20	20	M	ME	41		Moderate	-
**161**	26/05/20	16.5	F	ME	38.5		None	-
**162**	26/05/20	18.4	M	ME	41		Moderate	-
**163**	26/05/20	17.5	F	ME	40		None	-
**164**	26/05/20	18.3	M	ME	42		None	-
**165**	26/05/20	21.5	M	ME	44		Moderate	-
**166**	26/05/20	18.9	F	ME	39.5		Moderate	-
**167**	26/05/20	18.3	M	ME	43		Moderate	-
**168**	26/05/20	17.8	F	ME	39	Early	Marked	-
**169**	26/05/20	17.9	M	ME	44		None	-
**170**	26/05/20	18.8	F	ME	42		Marked	-
**171**	26/05/20	18.4	M	ME	42		Marked	-
**172**	26/05/20	17.7	M	ME	42		Mild	-
**173**	26/05/20	17.5	F	ME	40		Mild	-
**174**	26/05/20	19	M	ME	43		Moderate	-
**175**	26/05/20	17.1	F	ME	40		Mild	-
**176**	26/05/20	20	M	ME	42		Marked	-
**177**	26/05/20	22.1	F	ME	40.5		Marked	-
**178**	26/05/20	18.6	M	ME	42		Severe	-

**179**	26/05/20	18.6	F	ME	40	Late	None	-
**180**	26/05/20	19.2	F	ME	40.5	Mid	None	-
**181**	26/05/20	25.1	F	ME	40.5	Late	Marked	-
**182**	26/05/20	18	F	ME	40	Early	Mild	-
**183**	26/05/20	20.6	F	ME	41	Mid	Mild	-
**184**	26/05/20	22.1	F	ME	40		Mild	-
**185**	26/05/20	18.3	M	ME	43		Moderate	-
**186**	26/05/20	20	F	ME	40.5	Mid	None	-
**187**	26/05/20	21.1	M	ME	43		Marked	-
**188**	26/05/20	18.1	F	ME	39.5	Mid	Moderate	-
**189**	26/05/20	16.5	M	ME	44		Moderate	-
**190**	26/05/20	17.5	F	ME	41		Mild	-
**191**	26/05/20	17.9	M	ME	43		Moderate	-
**192**	26/05/20	20.3	F	ME	41	Late	Mild	-
**193**	26/05/20	17.2	F	ME	42	Early	Mild	-
**194**	26/05/20	23.9	F	ME	41.5	Late	Marked	-
**195**	26/05/20	17.3	F	ME	41	Late	None	-
**196**	26/05/20	19.6	F	ME	40.5	Mid	None	-
**197**	26/05/20	20.6	F	ME	45	Mid	Severe	-
**198**	26/05/20	17.2	F	ME	43		None	-
**199**	26/05/20	18.4	F	ME	42	Mid	None	-
**200**	26/05/20	18.8	F	ME	42		None	-
**201**	01/06/20	21	F	ME	43		Moderate	-
**202**	01/06/20	15.6	F	ME	39		Mild	-
**203**	01/06/20	18.8	M	ME	39.5		Marked	-
**204**	01/06/20	18.9	M	ME	40		None	-
**205**	01/06/20	20.6	F	ME	40	Late	Marked	-
**206**	01/06/20	20.9	F	ME	42	Late	Marked	-
**207**	01/06/20	16.7	F	ME	40		None	-
**208**	01/06/20	15.8	F	ME	39.5		Moderate	-
**209**	01/06/20	17.6	F	ME	42	Mid	Marked	-
**210**	01/06/20	18	M	ME	39.5		None	-
**211**	01/06/20	16.1	F	ME	40		Mild	-
**212**	01/06/20	21.4	F	ME	40.5	Late	None	-
**213**	01/06/20	16.9	F	ME	42	Mid	Mild	-
**214**	01/06/20	18.2	F	ME	40	Early	Marked	-
**215**	01/06/20	21	F	ME	41	Late	Moderate	-
**216**	01/06/20	19.2	M	ME	40.5		Mild	-
**217**	01/06/20	16.8	F	ME	41.5		Mild	-
**218**	01/06/20	17.2	F	ME	40.5		Moderate	-
**219**	01/06/20	22	F	ME	40.5	Late	None	-
**220**	01/06/20	18.2	M	ME	40.5		Moderate	-
**221**	01/06/20	18.8	M	ME	39.5		Marked	-
**222**	01/06/20	18	F	ME	41	Mid	Mild	-
